# Enhanced Oncolytic Potential of Engineered Newcastle Disease Virus Lasota Strain through Modification of Its F Protein Cleavage Site

**DOI:** 10.3390/microorganisms12102029

**Published:** 2024-10-08

**Authors:** Zedian Li, Weifeng Qian, Yuhao Zhang, Chengshui Liao, Jian Chen, Ke Ding, Qingzhong Yu, Yanyan Jia, Lei He

**Affiliations:** 1 The Key Lab of Animal Disease and Public Health/Luoyang Key Laboratory of Live Carrier Biomaterial and Animal Disease Prevention and Control, Henan University of Science and Technology, Luoyang 471023, China; lizedian@126.com (Z.L.); qwf2012@yeah.net (W.Q.); yuhaozhang19991107@163.com (Y.Z.); liaochengshui33@haust.edu.cn (C.L.); chenillejian@163.com (J.C.); keding19@163.com (K.D.); 2 Southeast Poultry Research Laboratory, US National Poultry Research Center, Agricultural Research Service, United States Department of Agriculture, Athens, GA 30605, USA; qingzhong.yu@usda.gov

**Keywords:** Newcastle disease virus, fusion protein cleavage site, oncolytic effect, murine melanoma, B16F10

## Abstract

Newcastle disease virus (NDV) is an oncolytic virus whose F protein cleavage activity is associated with viral infectivity. To explore the potential of modifying F protein cleavage activity to enhance antitumor effects, we constructed a recombinant NDV LaSota strain by replacing its F protein cleavage site with that from the mesogenic Beaudette C (BC) strain using reverse genetics techniques. The resulting virus, rLaSota-BC-RFP, demonstrated significantly enhanced infectivity and tumor cell suppression on the murine melanoma B16F10 cell, characterized by higher cytotoxicity and increased apoptosis compared to its parental strain, rLaSota-RFP. In vivo, rLaSota-BC-RFP treatment of B16F10 tumors in C57BL/6 mice resulted in significant tumor growth inhibition, improved survival rate, and induction of tumor-specific apoptosis and necrosis. Additionally, the rLaSota-BC-RFP treatment enhanced immunostimulatory effects within the tumor microenvironment (TME), characterized by increased infiltration of CD4^+^ and CD8^+^ T cells and elevated levels of antitumor immune modulator cytokines, including mouse IL-12, IFN-γ, IL-15, and TNF-α, in the rLaSota-BC-RFP-treated tumor tissues. Collectively, these findings demonstrate that the mesogenic F protein cleavage site enhances the oncolytic potential of the NDV LaSota strain, suggesting that rLaSota-BC-RFP is a promising oncolytic viral vector for gene delivery in cancer immunotherapy.

## 1. Introduction

Cancer ranks as the first or second leading life-threatening disease worldwide, accounting for nearly 10 million deaths in 2020 based on the reported data [1]. While traditional treatments like surgery, radiation, and chemotherapy remain crucial, innovative approaches like oncolytic virus therapy offer promising alternatives with potentially fewer side effects.

Newcastle disease virus (NDV) is a well-studied oncolytic virus that has shown promise in preclinical animal models against various cancers, including lung, breast, prostate, colorectal, glial, and melanoma [2,3,4,5]. This virus has been found to exert its oncolytic efficacy through three key mechanisms. Firstly, NDV selectively replicates in human cancer cells at higher levels than normal cells. Clinical trials have demonstrated its ability to replicate effectively in tumors without harming healthy tissues. Secondly, NDV can induce both intrinsic and caspase-dependent apoptosis in cancer cells [6,7]. Finally, growing evidence shows that NDV could activate the immune system, triggering both innate and adaptive antitumor immune responses [8,9].

NDV is a member of the genus *Orthoavulavirus* within the subfamily *Avulavirinae* of the family *Paramyxoviridae* [10]. NDV possesses a single-stranded, non-segmented, negative-sense RNA of 15186, 15192, or 15198 nucleotides in length. Its genome, flanked by a 3′ leader and a 5′ trailer, contains six genes arranged in the following order: nucleocapsid protein (NP), phosphoprotein (P), matrix protein (M), fusion protein (F), hemagglutinin-neuraminidase (HN), and large polymerase (L) [11]. NDV isolates can be classified into three groups based on their virulence in birds: (1) lentogenic strains, (2) mesogenic stains, and (3) velogenic strains. The lentogenic strains are generally considered non-lytic. In contrast, velogenic and mesogenic strains are lytic, capable of replicating and producing progeny virus within cancer cells, leading to cell destruction [12]. The infectious progeny produced by lytic viruses can facilitate subsequent rounds of viral virus replication within the tumor tissues, leading to a multi-cycle infection process [13]. The LaSota strain is a lentogenic NDV strain, generally considered non-lytic. However, some studies have suggested it may exhibit oncolytic properties in certain cancer cells [14,15]. The LaSota strain efficiently replicates in primary chicken embryo fibroblast (CEF) cells and the DF-1 cell line. However, its growth in tumor cells may be constrained due to its F protein cleavage site. This site lacks the furin motif, which is essential for intracellular proteolytic cleavage. As a result, the LaSota strain relies on extracellular proteases for cleavage, potentially limiting its replication activity within tumor cells [16]. Velogenic and mesogenic strains possess a cleavage site that can be recognized by furin-like proteases, activating the F glycoprotein in a wide range of tumor cell types. This activation facilitates efficient viral replication and allows for the spread of the virus from one cell to another [17].

Reverse genetics technology has enabled the precise modification of NDV genomes, making it a versatile platform for various applications, including oncolytic viral vectors for cancer therapy. In this study, using reverse genetics, we modified the LaSota strain of NDV to improve its oncolytic activity. We replaced the F protein cleavage site of the LaSota strain with that of the mesogenic Beaudette C (BC) strain in the pLaSota-RFP infectious clone [18]. The red fluorescent protein (RFP) gene inserted between the P and M genes in the infectious clone was used as a reporter to track viral dissemination and monitor its oncolytic efficiency on tumor cells. RFP is widely used in oncolytic virus research and has minimal impact on cell viability and function. The resulting engineered LaSota recombinant, rLaSota-BC-RFP, demonstrated enhanced oncolytic activity compared to its parental virus, rLaSota-RFP, in both in vitro and in vivo murine melanoma B16F10 cell models. These findings suggest that rLaSota-BC-RFP is a promising viral delivery vector for cancer immunotherapy research.

## 2. Materials and Methods

### 2.1. Cell Lines, Animals, and SPF Eggs

The cell lines B16F10 (CRL-6475; ATCC), BHK-21(CCL-10; ATCC), U251(09063001; ECACC), and DF-1(CRL-3586; ATCC) were cultured in high-glucose Dulbecco’s modified Eagle medium (DMEM, Biosharp, Hefei, China), supplemented with 10% fetal bovine serum (FBS, CLARK, Shanghai, China) and antibiotics (100 U/ml Penicillin, 100 µg/ml Streptomycin, and 0.25 µg/mL Amphotericin B, Servicebio, Wuhan, China). The cultures were maintained at 37 °C in a 5% CO_2_ incubator. The modified vaccinia Ankara/T7 recombinant virus (MVA/T7), which provides the bacteriophage T7 RNA polymerase for virus rescue, was kindly provided by B. Moss from the National Institutes of Health [19].

Six-week-old female C57BL/6 mice were purchased from Zhengzhou Jiacheng Biotechnology Co. (Zhengzhou, China) and acclimatized to their new environment for one week. They were housed in a pathogen-free, temperature-controlled environment. Animal experiments were conducted in compliance with the guidelines issued by the Experimental Animal Commission of Henan University of Science and Technology (Permit No. SCXK [YU] 2020–0001) and the Institutional Animal Care and Use Committee (No. 201) of Henan University of Science and Technology (Luoyang, Henan, China). Specific pathogen-free (SPF) chicken embryos were obtained from Merial Vital Laboratory Animal Technology Co., Ltd. (Beijing, China) and incubated at 37 °C with 70% relative humidity.

### 2.2. Construction and Rescue of Recombinant rLaSota-BC-RFP

The previously generated pLaSota-RFP infectious clone [18] served as a backbone for constructing the LaSota strain-modified infectious clone containing the F protein cleavage site from the mesogenic Beaudette C (NDV-BC) strain. The LaSota-RFP vector was amplified by PCR with *pfu* Ultra^TM^ II Fusion HS DNA polymerase (Agilent Technologies, La Jolla, CA, USA) and paired gene-specific primers (LaSota-BC-RFP SITE F: AGGAGACAGAAACGCcttgagacaaagccgtcaacat; LaSota-BC-RFP SITE R: GCGTTTCTGTCTCCTcagctgcaagaggcctgccatc) according to the manufacturer’s instructions. The capital letters in the PCR primers represent the BC strain’s F protein cleavage site sequences. Subsequently, the amplified laSota-RFP PCR product was self-ligated using an In-Fusion^®^ PCR Cloning Kit (TaKaRa Bio Inc., Dalian, China). The resulting recombinant plasmid, designated as pLaSota-BC-RFP, was propagated in Stbl2 cells (WEIDI Bio Inc., Shanghai, China) at 30 °C for 18 h and purified using a High Pure Plasmid Miniprep Kit (Beijing Zoman Biotechnology Co., Ltd., Beijing, China).

The recombinant virus was rescued by co-transfecting the recombinant cDNA clone pLaSota-BC-RFP (2 µg) with the helper plasmids pLs-NP (1 µg), pLs-P (0.5 µg), and pLs-L (0.25 µg) into MVA/T7 virus pre-infected BHK-21 cells using lipofectamine 8000 (Beyotime Biotechnology Co., Ltd. Shanghai, China) according to manufacturer’s instruction. At 72 h post transfection, the transfected cells were harvested by freeze–thawing and injected into the allantoic cavities of 9-day-old embryonated SPF chicken eggs to amplify the rescued virus. After 4 days post incubation, the allantoic fluids (AF) were harvested and detected by the hemagglutination (HA) assay. HA-positive AF was filtered twice through a 0.22 µm filter and propagated in embryonated SPF chicken eggs for 2 passages. The AF was harvested, aliquoted, and stored at−80 °C as a stock.

### 2.3. Biological Characterization of the Recombinant Virus rLaSota-BC-RFP

The recombinant virus rLaSota-BC-RFP and its parental rLaSota-RFP virus were titrated using the standard HA test, the 50% egg infective dose (EID_50_) assay in 9-day-old SPF chicken embryos. The pathogenicity of the recombinant viruses was determined using the standard mean death time (MDT) assays in embryonated SPF chicken eggs [20].

The growth kinetics of the recombinant NDVs were determined using a growth assay with DF-1 cells. Briefly, DF-1 cells were infected with each virus at a multiplicity of infection (MOI) of 0.01. The infected DF-1 cells were harvested at 24 h intervals and stored at −80 °C. Virus titers at each time point were determined by TCID_50_ assay on DF-1 cells in triplicate [20]. The viral titer is expressed in Log_10_ TCID_50_/mL. The complete genomic sequence of the rescued virus was determined by sequencing the RT-PCR products amplified from the virus genome to confirm the sequence fidelity of the recombinant viruses.

### 2.4. The RFP Expression and Morphological Alterations of B16F10 Cells

The B16F10 and human malignant glioblastoma multiforme (GBM) U251 cells were seeded at a density of 3 × 10^5^ cells in a 24-well plate and infected with 0.1 MOI of rLaSota-RFP and rLaSota-BC-RFP, respectively. The infected B16F10 cells were observed and digitally photographed using an inverted fluorescence microscope at 100 × magnifications to record the morphological changes at 24 h intervals (ZEISS, Axio Observer, Oberkochen, German) for 3 days.

### 2.5. Cell Proliferation and Viability Assay

Cell proliferation and viability were detected using a Cell Counting Kit-8 kit (Beyotime Biotechnology Co., Ltd. Shanghai, China). Briefly, B16F10 and U251 cells were seeded at a density of 5 × 10^4^ cells into 96-well plates. Subsequently, the cells were infected with rLaSota-RFP and rLaSota-BC-RFP at 0.1 MOI, respectively. At 24, 48, and 72 h post infection, 10 µl of CCK-8 solution was added to each well and then incubated for 2 h. The absorbance was measured with a wavelength of 450 nm using a spectrophotometer. All the experiments were repeated in triplicates independently. The cell viability was converted and expressed using the formula:

Cell viability = (NDV infected group OD − cell control group OD)/(cell control group OD) × 100%

### 2.6. Wound Healing Assay

B16F10 cells were seeded into 6-well plates. A sterile 200 µl microtip was used to generate a horizontal scratch as an artificial wound at 24 h post culture. After being washed twice with PBS to remove the floating and dead cells, the cells were infected with 0.1 MOI rLaSota-RFP and rLaSota-BC-RFP, respectively. Cell migrations in the denuded area were observed and photographed using an inverted microscope at the indicated time points. The migrated distance was deemed to represent the migration rate. This assay was carried out in triplicate.

### 2.7. Western Blot Assay

B16F10 cells were infected with rLaSota-RFP and rLaSota-BC-RFP at a MOI of 0.1. Following incubation for 12 h, the infected cells were lysed, and their protein concentration was determined using a BCA kit (TaKaRa Bio Inc, Dalian, China). Equal amounts of protein (20 µg) were separated by SDS-PAGE and transferred to PVDF membranes. The membranes were then probed with rabbit anti-NDV-F pAb (1:500, provided by Professor XuSheng Qiu), anti-Caspase-3 rabbit pAb (1:1000), anti-Caspase-9 rabbit pAb (1:800, Servicebio Biotechnology Co., Ltd., Wuhan, China), or GAPDH rabbit mAb (1:1000), respectively. HRP-conjugated goat anti-rabbit IgG (1:1000) was used as the secondary antibody. Protein bands were detected with the BeyoECL Plus system (Beyotime Biotechnology Co., Ltd. Shanghai, China), and the image was captured by the Chemiluminescence Apparatus (Tanon Biotechnology Co., Ltd., Shanghai, China).

### 2.8. Animal Experiments

To assess the oncolytic efficacy of rLaSota-BC-RFP and rLaSota-RFP in vivo, 1 × 10^5^ B16F10 cells were subcutaneously implanted into six-week-old female C57BL/6 mice. Tumor growth was monitored every two days. When tumors reached 50 mm^3^, the mice were randomly divided into three groups (*n* = 15). Each group received intratumoral injections of 100 µl of rLaSota-RFP (3 × 10^7^ EID_50_/mL), rLaSota-BC-RFP (3 × 10^7^ EID_50_/mL) or PBS, respectively, every two days for a total of 8 injections. Tumor volume was calculated using the formula V = (*ab*^2^)/2, where *a* is the long axis, and *b* is the short axis of the tumor. The mice were monitored daily and euthanized when the tumor size reached 2000 mm^3^ or at the end of the experiment.

At 16 days post infection, all control animals and three mice from each treatment group were euthanized. The tumors were then excised for immunochemistry and real-time qPCR assays. The remaining mice were observed for 25 days with tumor volume monitoring to determine survival rates.

### 2.9. Histological Analysis and TUNEL Assay

Mouse tumor samples were fixed in 4% paraformaldehyde, embedded in paraffin, sectioned at 3 μm, and stained with hematoxylin-eosin (H&E). TUNEL assays (terminal deoxynucleotidyl transferase-mediated dUTP nick and labeling assay) were performed to assess apoptosis. Additionally, tumor-infiltrating CD8^+^ T cells and CD4^+^ T cells were detected in the tumor samples using anti-CD8 and anti-CD4 antibodies (Servicebio, Wuhan, China). The mean fluorescence intensity of green fluorescence was measured using Image J 1.54 software. All assays were conducted in triplicate.

### 2.10. Real-Time Quantitative PCR (RT-qPCR)

To investigate the expression of apoptosis-related genes and immune regulator proteins, total RNA was isolated from the NDV-infected cells and separate mouse tumor samples from each treatment group using a Total RNA Extraction Kit RNA fast 2000 remover (Feijie Biotechnology Co., Ltd., Wuhan, China) and reverse-transcribed with the M5 Super plus qPCR RT kit with a gDNA remover (Mei5bio Biotechnology Co., Ltd., Beijing, China) according to the manufacturers’ protocols. RT-qPCR for IL-15, IL-12, tumor necrosis factor alpha (TNF-α), Interferon gamma (IFN-γ), vascular endothelial growth factor A(VEGF-A), and epidermal growth factor receptor (EGFR) were performed using the M5 HiPer Realtime PCR mix (Mei5bio Biotechnology Co., Ltd., Beijing, China) in a 7500 Fast Real-Time qPCR System (Bio-Rad, Hercules, CA, USA) with GAPDH as the reference gene. Primer sequences are shown in Supplementary Table S1. The relative gene expression was quantified using the 2^−△△Ct^ method, and data analysis was performed using the CFX ManagerTM software Version 3.1. The results are expressed as fold changes in relative mRNA expression levels compared to the PBS group.

### 2.11. Statistical Analysis

Statistical analysis was conducted using the GraphPad Prism 8.02 software. Data are presented as the mean ± standard deviation. Unpaired student’s *t*-test or one-way analysis of variance (ANOVA) was employed to analyze the data. Statistical significance was determined using *p*-values: * *p* < 0.05, ** *p* < 0.01, *** *p* < 0.001.

## 3. Results

### 3.1. Generation and Growth Curve of the Recombinant NDVs

**Figure 1:**
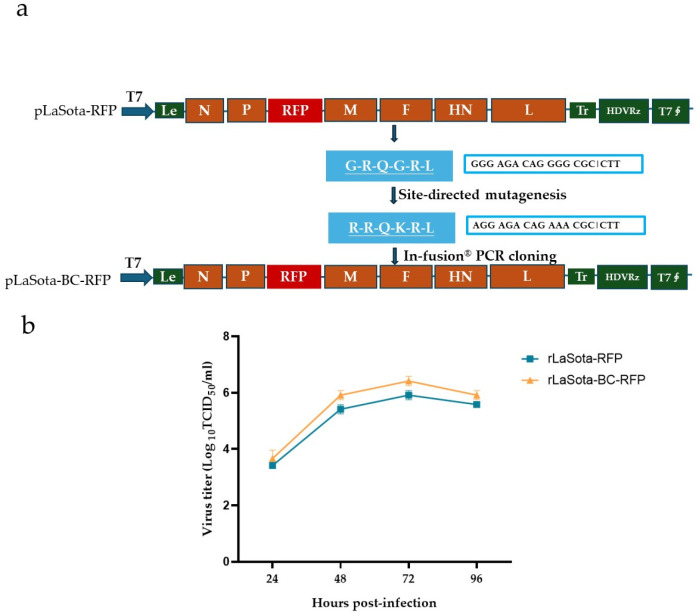
Generation and growth curve of the recombinant NDVs. (**a**) Schematic representation of pLaSota-BC-RFP construction. The F protein cleavage site sequence in the previously generated infectious clone, pLaSota-RFP [18], was replaced with that of the NDV BC strain using an In-Fusion^®^ PCR Cloning Kit (TaKaRa Bio Inc., Dalian, China). (**b**) Growth curve of recombinant viruses. DF-1 cells were infected with the indicated NDV viruses at 0.01 MOI. Virus lysates were harvested every 24 h post infection and titrated on DF-1 cells using the TCID_50_ titration method. Virus titers are expressed as mean log_10_ TCID_50_/mL with standard deviation.

A full-length cDNA clone of the recombinant NDV LaSota strain incorporating the mesogenic Beaudette C (BC) strain F protein cleavage sequence and the red fluorescence protein (RFP) gene was constructed using PCR and In-Fusion^®^ PCR cloning (Figure 1a). The modified F protein addresses the limitation of the parental LaSota strain, which requires additional extracellular protease for infectivity activation. RFP serves as a reporter to track the virus growth and the oncolytic activity of NDV in B16F10 cells. Additionally, it could potentially be replaced by other antitumor proteins, expanding the therapeutic applications of this modified recombinant virus. The recombinant virus, rLaSota-BC-RFP, was successfully generated by cotransfecting the full-length cDNA clone and supporting plasmids into BHK-21 cells, followed by propagation in SPF embryonated chicken eggs. The nucleotide sequence fidelity of the recombinant virus was confirmed by sequencing analysis of the RT-PCR products from the viral genome.

The growth assay revealed that the rLaSota-BC-RFP virus replicated in DF-1 cells with a slightly higher titer than its parental rLaSota-RFP virus but without significantly altering the growth kinetics (Figure 1b). As shown in Table 1, the rLaSota-BC-RFP virus slightly increased its pathogenicity, with a shorter MDT and a one log lower virus titer in embryonated chicken eggs compared to rLaSota-RFP. These findings suggest that the modified F protein cleavage site affected viral infectivity and pathogenicity.

### 3.2. The rLaSota-BC-RFP Recombinant Increased the Inhibition of B16F10 Cell Growth In Vitro

**Figure 2:**
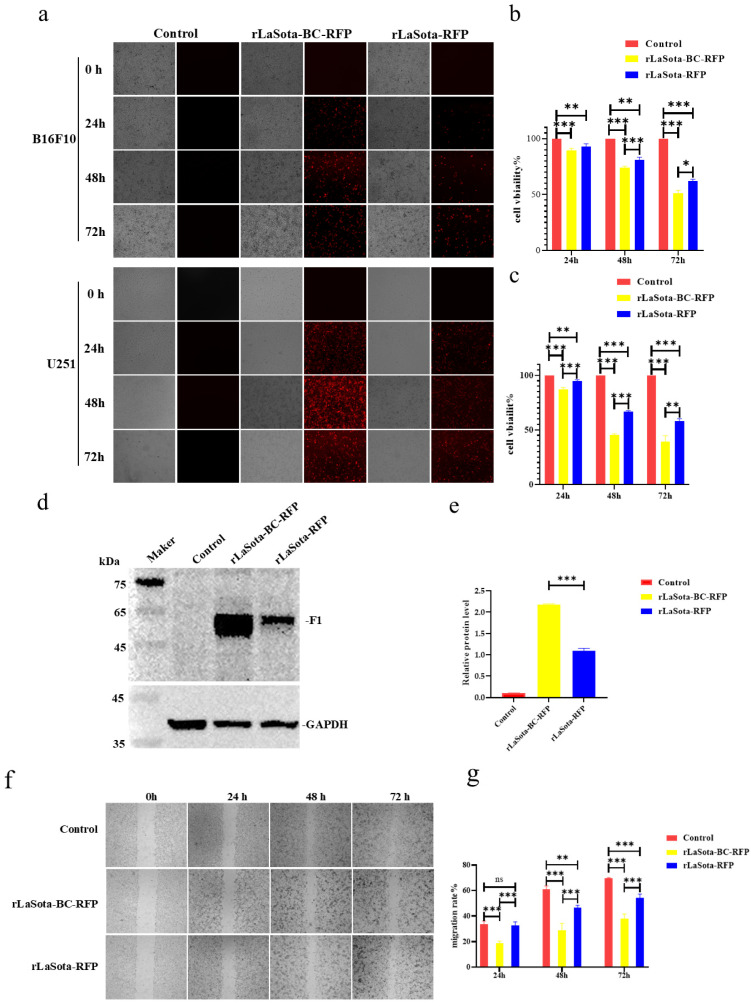
The LaSota recombinant with a mesogenic F protein cleavage site increased the inhibition of tumor cell growth in vitro. (**a**) B16F10 and U251 cells were infected with the indicated recombinant viruses at 0.1 MOI. At 24, 48, and 72 h post infection, infected cells were examined under an inverted fluorescence microscope at 100 × magnifications. RFP was observed using a 532 nm excitation wavelength filter and photographed for preservation. (**b**,**c**) The cytotoxicity of recombinant viruses in tumor cells. B16F10 (**b**) and U251 (**c**) cells were infected with the indicated recombinant viruses at 0.1 MOI. At 24, 48, and 72 h post infection, cell viability was determined by the CCK-8 assay (mean ± SD). (**d**,**e**) The relative protein expression of NDV F protein and GAPDH protein in B16F10 cells infected with recombinant viruses at 0.1 MOI, 24 h. (**f**) Cell migration post infection. Confluent monolayers of B16F10 cells were infected with 0.1 MOI of rLaSota-BC-RFP or rLaSota -RFP. The migratory distance of the infected cells was measured and photographed at 0, 24, 48, and 72 h post infection. (**g**) A comparison of cell migration rates. B16F10 cell migration rates were calculated based on the cell migration distance post infection. Cell migration rates among the infected and control cells were compared. Data are plotted as mean ± SD with statistical significance: * *p* < 0.05, ** *p* < 0.01, *** *p* < 0.001.

To evaluate the impact of the F protein cleavage site on LaSota recombinant oncolytic ability in tumor cells, we initially measured the infection efficiency and growth inhibition capacity in two representative tumor cells: B16F10 and U251. As shown in Figure 2a, both rLaSota-RFP and rLaSota-BC-RFP replicated effectively within the cell lines. The number of fluorescent cells and the RFP fluorescence intensity peaked approximately 48 h post-infection. Notably, more fluorescent cells were observed in the rLaSota-BC-RFP-infected cells than in rLaSota-RFP-infected cells, indicating that the BC F protein cleavage site enhanced virus replication in tumor cells. To further explore the cytotoxic effects of the LaSota recombinants on tumor cells, we measured the viability of virus-infected B16F10 and U251 cells using CCK-8 assays. As shown in Figure 2b,c, the LaSota recombinants exhibited a marked pronounced growth inhibitory effect as the infection progressed. Specifically, B16F10 and U251 cells infected with 0.1 MOI of rLaSota-BC-RFP exhibited a dramatic decrease in viability to 51.35% and 41.65%, respectively, at 72 h post-infection. Statistically, rLaSota-BC-RFP virus induced a significantly more potent cytotoxic effect on the tumor cell line than rLaSota-RFP.

To further elucidate the impact of the F cleavage site mutation on NDV replication within B16F10 cells, we conducted Western blot analysis to assess the cleavage status of the NDV F protein (Figure 2d,e). Cells infected with rLaSota-BC-RFP demonstrated a substantial two-fold increase in cleaved F1 protein expression compared to those infected with rLaSota-RFP. This finding strongly suggests that the F cleavage site replacement significantly enhanced F protein cleavage efficiency, improving viral infectivity on B16F10 cells. Additionally, the scratch assay revealed that the infection of B16F10 cells with rLaSota-BC-RFP or rLaSota-RFP inhibited cell migration (Figure 2f,g). At 72 h post infection, the migration rate of B16F10 cells infected with 0.1 MOI of rLaSota-BC-RFP or rLaSota-RFP dropped to 52.41% ± 2.10% and 66.25% ± 2.33%, respectively (Figure 2e). Combined with the previous results, these findings strongly suggest that the F cleavage site mutation enhances NDV infection and tumor cell growth inhibition.

### 3.3. The rLaSota-BC-RFP Recombinant Enhanced Apoptosis of B16F10 Cells In Vitro

**Figure 3:**
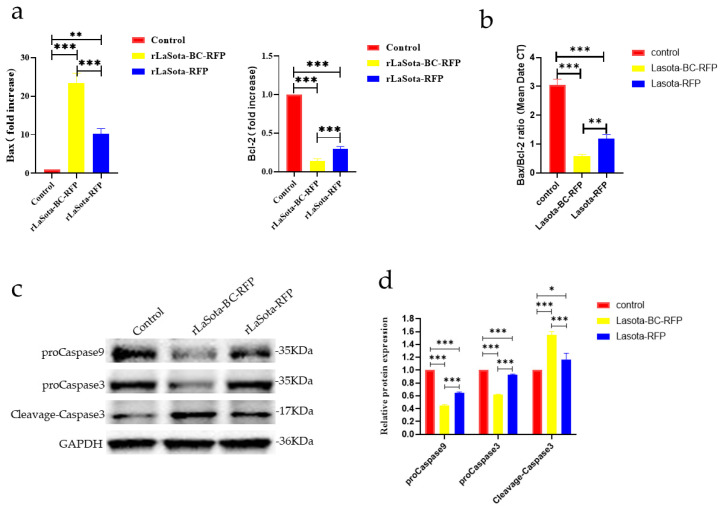
The impact of recombinant viruses on the induction of tumor cell apoptosis. (**a**) The transcriptional levels of Bax and Bcl-2 genes in B16-F10 cells were calculated relative to the GAPDH. Both the cells were infected with recombinant viruses at an MOI of 0.1 for 12 h. (**b**) The ratio of Bax/Bcl-2 mRNA expression in control and recombinant virus-infected cells by RT-qPCR. (**c**) The relative protein expression of proCaspase3, cleaved-Caspase3, and proCaspase9 in B16F10 cells infected with recombinant viruses at 0.1 MOI, 12 h. (**d**) The relative intensities of proCaspase3, cleaved-Caspase3, and proCaspase9 were quantified by ImageJ software, v 1.54. GAPDH was used as a normalized control. Data are plotted as mean ± SD with statistical significance: * *p* < 0.05, ** *p* < 0.01, *** *p* < 0.001.

The capability of recombinant viruses to induce apoptosis in tumor cells was investigated. Two key proteins, Bax and Bcl-2, involved in the regulation of apoptosis, were analyzed using the RT-qPCR method; the results showed that recombinant virus infection upregulated Bax and downregulated Bcl-2 in B16F10 cells, indicating an increased propensity for apoptosis (Figure 3a,b). Furthermore, both viruses increased cleaved caspase-3 protein while decreasing procaspase-3 and -9. However, rLaSota BC-RFP exhibited a more pronounced effect on procaspase-3, -9, and cleaved caspase-3 levels, suggesting that its modified F cleavage site accelerates tumor cell apoptosis. Overall, these results demonstrate that the modified F protein in rLaSota BC-RFP enhances its ability to induce apoptosis in tumor cells.

### 3.4. Recombinant NDVs Significantly Inhibited Tumor Growth in the B16F10 Mouse Model

**Figure 4:**
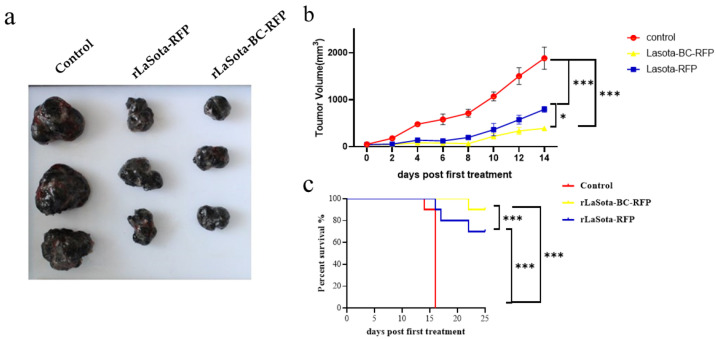
The antitumor effects of recombinant LaSota viruses on B16F10 melanoma. (**a**) The macroscopic appearance of tumors excised from mice treated with rLaSota-RFP or rLaSota-BC-RFP and PBS-treated control mice (*n* = 3) at 16 days post treatment. (**b**) The tumor growth curves of mice treated with rLaSota-BC-RFP, rLaSota-RFP, or PBS during the first 14 days post treatment. (**c**) Survival rates of tumor-bearing mice treated with rLaSota-BC-RFP rLaSota-RFP, or PBS, over 25 days after the first treatment (*n* = 10). Data were analyzed using variance (ANOVA) (**c**). * *p* < 0.05 *** *p* < 0.001.

To validate the in vitro tumor cell growth inhibition observed with recombinant NDVs, we conducted in vivo experiments using the B16F10 mouse model. Female C57BL/6 mice bearing B16F10 melanoma tumors were treated with recombinant viruses intratumorally. Tumor volumes were monitored, and three tumor tissues were excised from the treated mice at 16 days post treatment (Figure 4a,b). Mice treated with rLaSota-RFP and rLaSota-BC-RFP exhibited average tumor sizes of 569.71 ± 76.50 mm^3^ and 315.42 ± 20.77 mm^3^, respectively. In contrast, the PBS control group had an average tumor size of 1859.52 ± 129.88 mm^3^, approximately 3–6 times larger than the virus-treated tumors. These results indicate that both recombinant viruses effectively inhibited tumor growth. Notably, rLaSota-BC-RFP-treated tumors were smaller (approximately 1.8-fold) than those treated with rLaSota-RFP. This suggests that the enhanced BC F protein cleavage site played a significant role in improving antitumor efficacy. Survival analysis further demonstrated the therapeutic advantage of rLaSota-BC-RFP. Additionally, as shown in Figure 4c, 90% of the mice treated with rLaSota-BC-RFP survived at the end of the experiment (25 days post treatment). In contrast, only 70% of mice treated with rLaSota-RFP survived. These findings collectively indicate that rLaSota-BC-RFP is a promising therapeutic candidate vector for B16F10 melanoma, demonstrating superior oncolytic activity and improved survival rates compared to the unmodified virus.

### 3.5. Recombinant NDVs Induced Tumor Necrosis and Apoptosis in the B16F10 Model

**Figure 5:**
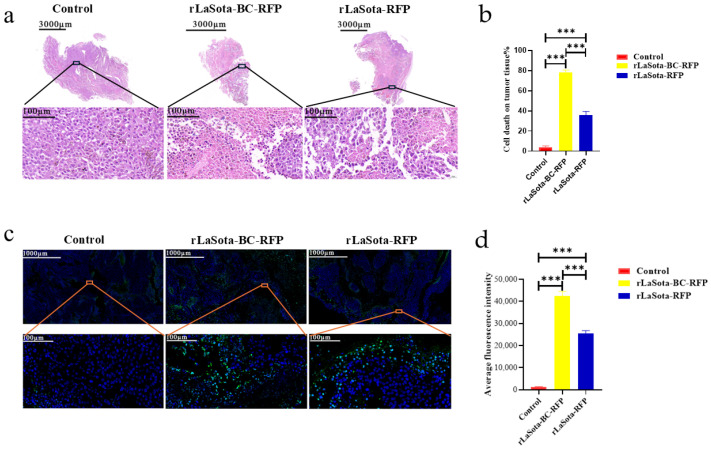
Effects of LaSota recombinant viruses on tumor necrosis and apoptosis in the B16F10 model. (**a**) H&E staining of tumors excised from mice treated with rLaSota-BC-RFP, rLaSota-RFP, or PBS at day 16 post treatment. The top row shows whole tumor sections, while the bottom row depicts magnified tumor bed areas. (**b**) The percentage of necrosis cells was quantified using ImageJ 1.54 software. (**c**) TUNEL assay to detect apoptotic cells (green) in tumor tissues. Nuclei were stained with DAPI (blue). (**d**) Quantification of apoptotic cells using ImageJ 1.54 software. The top row shows whole tumor sections, while the bottom row depicts magnified tumor bed areas. Data were analyzed using the variance (ANOVA). *** indicates *p* < 0.001.

To assess tumor necrosis induced by different treatments, we performed H&E staining on tumors excised from PBS-treated control mice and mice treated with rLaSota-RFP or rLaSota-BC-RFP. As shown in Figure 5a, PBS-treated tumors displayed well-organized tumor cells with regular nuclei and prominent nucleoli. In contrast, rLaSota-RFP-treated tumors exhibited nuclear pyknosis and loss of nucleoli in necrotic regions. rLaSota-BC-RFP-treated tumors showed even more extensive necrosis, characterized by nuclear deformation, pyknosis, and indistinct nuclear structures. Image J analysis revealed a significantly higher percentage of necrotic tumor cells in the rLaSota-BC-RFP group (78.25%) compared to the rLaSota-RFP group (35.67%). No necrosis was observed in the PBS control group (Figure 5b). These results demonstrate that rLaSota-BC-RFP induces more extensive tumor cell necrosis than rLaSota-RFP, highlighting its superior oncolytic potential. Furthermore, we performed TUNEL assays on tumor tissues to assess apoptosis induction by LaSota recombinants. Figure 5c,d demonstrate significant differences in TUNEL signal fluorescence among different treatment groups. Tumor tissues treated with recombinant NDVs exhibited a marked increase in apoptotic cells compared to the PBS-treated control group. Notably, the rLaSota-BC-RFP group displayed the highest rate of apoptosis. These findings further support the oncolytic effectiveness of LaSota recombinants, demonstrating their ability to induce apoptosis in tumor cells.

### 3.6. Treatment of Recombinant NDVs Led to Increased Immune Cell Infiltration and Immunomodulation within Tumor Tissues

**Figure 6:**
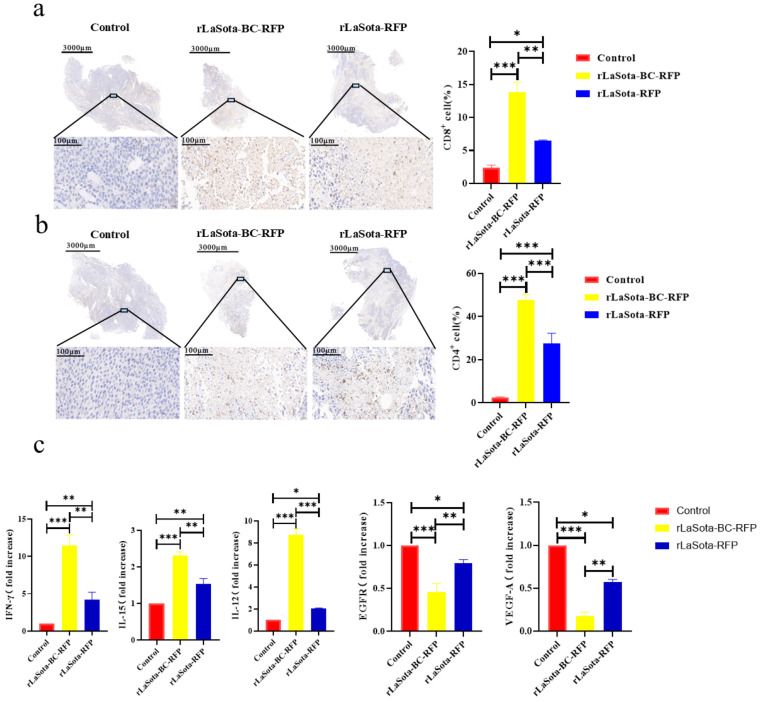
Immune cell infiltration and cytokine modulation in recombinant NDV-treated tumors. Immunohistochemical staining of CD4^+^ T cells (**a**) and CD8^+^ T cells (**b**) in the tumor microenvironment (brownish yellow). Nuclei were stained with hematoxylin (blue). The top row shows whole tumor sections, while the bottom row shows magnified tumor bed areas. Graphs display quantitative analysis of CD4^+^ (**a**) and CD8^+^ (**b**) cells (mean ± SD; *n* = 3). (**c**) RT-qPCR analysis of cytokine expression in tumors. Graphs show fold changes relative to the control (mean ± SD; *n* = 3). Data were analyzed using the two-tailed student t-test. * *p* < 0.05, ** *p* < 0.01, *** *p* < 0.001.

To investigate the immunostimulatory potential of recombinant NDVs in vivo, we examined the immune cell infiltration within the TME of B16F10-bearing mice. Immunohistochemistry was employed to detect CD4^+^ and CD8^+^ T cells in tumor tissues. As illustrated in Figure 6a,b, rLaSota-BC-RFP-treated tumors demonstrated significantly higher levels of tumor-infiltrating CD4^+^ (*p* = 0.008) and CD8^+^ (*p* = 0.0003) T lymphocytes compared to rLaSota-RFP- and PBS-treated groups in the B16F10 model. These findings suggest that the BC F protein cleavage site enhances the immunostimulatory capacity of the NDV LaSota strain, leading to increased immune cell infiltration within the TME. To determine whether the T cells recruited to the TME by recombinant NDVs possess antitumor properties, we analyzed changes in antitumor immune modulator cytokines (TNF-α, IFN-γ, IL-12, IL-15, VEGF-A, and EGFR) within the tumors. As shown in Figure 6c, compared to the PBS control and the rLaSota-RFP groups, the rLaSota-BC-RFP group exhibited significant increases in TNF-α (*p* = 0.037), IFN-γ (*p* = 0.001), IL-12 (*p* = 0.0004) and IL-15 (*p* = 0.0021) levels. Conversely, VEGF-A (*p* = 0.0049) and EGFR (*p* = 0.0474) levels were substantially decreased in the rLaSota-BC-RFP group. These findings suggest that the T cells recruited by rLaSota-BC-RFP are functionally active and capable of producing antitumor cytokines, contributing to its enhanced therapeutic efficacy.

## 4. Discussion

Oncolytic viruses (OVs) represent a promising class of natural immunotherapies. These viruses exert their oncolytic effects by infecting and replicating within tumor tissues. OVs can be either naturally existing viruses that have been weakened or genetically engineered. NDV has been recognized as an oncolytic virus since its anti-cancer and immune-boosting properties were first described in 1965 [21]. NDV offers several advantages as an ideal oncolytic virus for treating human and animal tumors. First, NDV is highly safe in humans due to host range restriction. A clinical oncolytic study reported no adverse effects following the intravenous administration of 10^¹⁰^ PFU NDV to humans [22]. Secondly, NDV selectively infects and replicates in tumor tissues while exhibiting minimal toxicity to normal tissues [23,24]. Furthermore, unlike other oncolytic viruses circulating in the human population, such as Adenovirus type 5 (Ad5) and Herpes Simplex Virus-1 (HSV-1), NDV does not elicit a pre-existing immune response in mammals, including humans. This lack of neutralizing antibodies eliminates a potential barrier to oncolytic virotherapy efficacy [25,26].

The LaSota strain is a naturally occurring lentogenic NDV strain that is safe for both animals and humans. However, it has limited oncolytic properties in certain cancer cells [14,15]. This is likely due to its F protein cleavage site sequence lacking the furin motif, which is essential for intracellular proteolytic cleavage for its replication within tumor cells [16]. To enhance the antitumor effects of the NDV LaSota strain, we generated a recombinant virus (rLaSota-BC-RFP) by replacing its F protein cleavage site with the highly fusogenic F protein cleavage site from the mesogenic Beaudette C strain using the reverse genetics. The antitumor efficacy of the recombinant virus was subsequently evaluated in the mouse B16F10 melanoma model. The cleavage of the F protein is known to be required for the initial step of NDV infection and is a major determinant of NDV virulence [12,27]. The multi-basic cleavage site allows for cleavage by a broad range of intracellular proteases, making the recombinant LaSota more effective in entering the tumor cells. in vitro and in vivo studies using the B16F10 melanoma model demonstrated that rLaSota-BC-RFP significantly outperformed its parental rLaSota-RFP virus in terms of antitumor activity. This enhancement can be attributed to the improved fusogenicity conferred by the modified F protein cleavage site.

Previous studies have reported that oncolytic NDV strains can exert their anticancer effects through caspase-dependent cell death pathways [28]. Cleaved caspase-3 activation has been observed in the early stages of NDV infection [29]. Zhang’s research shows that NDV can induce apoptosis in OSCC primarily via the mitochondrial pathway [30]. In this study, we detected a significant increase in cleaved caspase-3 levels in rLaSota-BC-RFP-infected cells at 12 h post-infection. These findings suggest that the anti-tumor effects of rLaSota-BC-RFP are associated with increased necrosis and proapoptotic processes in B16F10 cells.

In alignment with previous in vitro studies that alterations in the F protein cleavage site could influence NDV’s oncolytic effect [31], our in vivo data provide the first direct evidence that enhancing fusion protein cleavage can significantly improve NDV LaSota strain’s antitumor activity. rLaSota-BC-RFP demonstrated superior tumor-suppressive effects in B16F10-bearing mice. Tumor volumes were markedly reduced in mice treated with rLaSota-BC-RFP compared to those treated with rLaSota-RFP or PBS. Furthermore, the rLaSota-BC-RFP group maintained the highest survival rate among all groups.

NDV can trigger potent antitumor immune responses by exposing tumor-related antigens to the tumor microenvironment and convert “cold tumors” to “hot tumors” [32]. Levels of CD4^+^ and CD8^+^ tumor-infiltrating T cells often correlate with improved clinical outcomes in various cancers [33]. It has been reported that NDV’s therapeutic effect is associated with tumor-infiltrating lymphocytes (TILs) and tumor-specific responses mediated by activated CD8^+^ T cells, NK cells, macrophages, and the activation of type I IFN response [34]. In this study, we demonstrated that the infection of B16F10 tumor cells with the rLaSota-BC-RFP virus increased the infiltration of CD8^+^ and CD4^+^ T cells in the tumor tissues, accompanied by elevated levels of IL-12, IFN-γ, IL-15, TNF-α, and IL-18.

The elevation of these cytokines should lead to the activation and differentiation of T cells, B cells, and NK cells within the tumor microenvironment, further modulating antitumor immunity [34,35,36]. Notably, IFN-γ levels were significantly elevated in the rLaSota-BC-RFP group. IFN-γ has been shown to induce apoptosis of cancer cells and control tumor growth [37]. In contrast to the increase in immune cells and cytokines within the NDV-infected tumor tissues, the levels of the vascular endothelial growth factor (VEGF-A) and vascular endothelial growth factor receptor (EGFR) were reduced, particularly in the rLaSota-BC-RFP group. The decrease in these factors is believed to inhibit angiogenesis and promote the antitumor response [38].

Numerous studies have demonstrated the potential of NDV as a vector for delivering immunotherapeutic agents to cancer cells. NDVs expressing checkpoint inhibitors (anti-PD-1 and anti-PD-L1 antibodies) [39], super agonists (rNDV–anti-CD28 antibodies) [40], and immunocytokines (such as interleukin-2, IL7, and IL15) [41,42] have shown promising results in both in vitro and in vivo models, suggesting that NDV could be a valuable engineered oncolytic vector. Our data have conclusively proven that rLaSota-BC-RFP is a superior oncolytic-engineered vector. The RFP gene in this vector can be easily replaced with any potential anti-tumor proteins/factors, making it a promising platform for the future development of more effective oncolytic agents.

## 5. Conclusions

In summary, this study engineered an NDV LaSota recombinant, rLaSota-BC-RFP, bearing the mesogenic BC strain F protein cleavage site. The research compared the oncolytic properties of rLaSota-BC-RFP with its parental virus, rLaSota-RFP, in tumor cell cultures and the B16F10 mouse melanoma model. The results demonstrated that the increased F protein cleavage activity of the rLaSota-BC-RFP virus has enhanced oncolytic activity in both in vitro and in vivo models. Infection with rLaSota-BC-RFP resulted in improved suppression of tumor cell growth, migration, and induction of apoptosis. In the B16F10 mouse melanoma model, the treatment with rLaSota-BC-RFP led to increased immune cell infiltration, cytokine production within tumor tissues, and more effective tumor growth inhibition, resulting in a higher survival rate. These data demonstrate that the mesogenic F protein cleavage site significantly enhances the infectivity and antitumor activity of the NDV LaSota strain. This suggests that rLaSota-BC-RFP holds promise as a candidate for cancer therapy and could also be utilized as an oncolytic viral vector for delivering antitumor genes or factors to enhance cancer immunotherapy.

## Figures and Tables

**Table 1:** Biological assessments of the recombinant NDVs.

Virus	MDT ^1^	EID_50_/mL ^2^	HA ^3^
rLaSota-RFP	140 h	3.16 × 10^9^	2^8^
rLaSota-BC-RFP	74 h	6.81 × 10^8^	2^8^

^1^ MDT: Mean death time assay in embryonated chicken eggs. ^2^ EID_50_: The 50% infective dose assay in embryonated chicken eggs. ^3^ HA: Hemagglutination titer expressed in Log_2_.

## Data Availability

The original contributions presented in the study are included in the article/Supplementary Material, further inquiries can be directed to the corresponding authors.

## Supplementary Materials

The following supporting information can be downloaded at: https://www.mdpi.com/article/10.3390/microorganisms12102029/s1, Table S1: The primers used for RT-qPCR.
