# Supplementary material for: Enhanced Oncolytic Potential of Engineered Newcastle Disease Virus Lasota Strain through Modification of Its F Protein Cleavage Site
Source: Microorganisms. 2024 Oct 8;12(10):2029. doi: 10.3390/microorganisms12102029 (PMC11510066; doi:10.3390/microorganisms12102029)
Supplement: Supplementary file 1 [file microorganisms-12-02029-s001.zip › microorganisms-3232094-supplementary.pdf]

microorganisms

Supplement

Table S1. The primers used for RT-qPCR.

Gene	Primer	Primer Sequences (5′→3′)
IFN-γ	IFN-γ-F	GCTTTGCAGCTCTTCCTCATG
	IFN-γ-R	TCTTCCACATCTATGCCACTTGA
IL-12	IL-12-F	TGGTTTGCCATCGTTTTGCTG
	IL-12-R	ACAGGTGAGGTTCACTGTTTCT
TNF-α	TNF-α-F	AAGGGAGAGTGGTCAGGTTGCC
	TNF-α-R	CCTCAGGGAAGAGTCTGGAAAGG
IL-15	IL-15-F	ATGTTCATCAACACGTCCTGACT
	IL-15-R	GCAGCAGGTGGAGGTACCTTAA
EGFR	EGFR-F	GCCATCTGGGCCAAAGATACC
	EGFR-R	GTCTTCGCATGAATAGGCCAAT
VEGF-A	VEGF-A-F	TTCGTCCAACTTCTGGGCTC
	VEGF-A-R	ACAGCAGTAAAGCCAGGGTC
Bax	Bax-F	GGAGCAGCTTGGGAGCG
	Bax-R	AAAAGGCCCCTGTCTTCATGA
Bcl-2	Bcl-2-F	ACTTCGCAGAGATGTCCAGTCA
	Bcl-2-R	TGGCAAAGCGTCCCCTC
GAPDH	GAPDH-F	TGGTGAAGAAGGCATCTGAG
	GAPDH-R	TGCTGTTGAAGTCGCAGGAG
